# Single-Larva RNA Sequencing Identifies Markers of Copper Toxicity and Exposure in Early *Mytilus californianus* Larvae

**DOI:** 10.3389/fphys.2021.647482

**Published:** 2021-12-09

**Authors:** Megan R. Hall, Andrew Y. Gracey

**Affiliations:** Department of Biological Sciences, University of Southern California, Los Angeles, CA, United States

**Keywords:** biomarker, *Mytilus californianus*, copper, marker of exposure, marker of toxicity, transcriptomic (RNA-seq), single larval sequencing

## Abstract

One of the challenges facing efforts to generate molecular biomarkers for toxins is distinguishing between markers that are indicative of exposure and markers that provide evidence of the effects of toxicity. Phenotypic anchoring provides an approach to help segregate markers into these categories based on some phenotypic index of toxicity. Here we leveraged the mussel embryo-larval toxicity assay in which toxicity is estimated by the fraction of larvae that exhibit an abnormal morphology, to isolate subsets of larvae that were abnormal and thus showed evidence of copper-toxicity, versus others that while exposed to copper exhibited normal morphology. Mussel larvae reared under control conditions or in the presence of increasing levels of copper (3–15 μg/L Cu^2+^) were physically sorted according to whether their morphology was normal or abnormal, and then profiled using RNAseq. Supervised differential expression analysis identified sets of genes whose differential expression was specific to the pools of abnormal larvae versus normal larvae, providing putative markers of copper toxicity versus exposure. Markers of copper exposure and copper-induced abnormality were involved in many of the same pathways, including development, shell formation, cell adhesion, and oxidative stress, yet unique markers were detected in each gene set. Markers of effect appeared to be more resolving between phenotypes at the lower copper concentration, while markers of exposure were informative at both copper concentrations.

## Introduction

Heavy metal contamination of freshwater and marine water bodies is a long-recognized problem, especially in urban regions where industrial byproducts are high (Livingstone et al., 1992). Water quality criteria are determined by assessment of contaminant toxicity to common organisms in the affected ecosystem (EPA, 1995, 2016; E50 Committee, 2013). The standard assay for metal toxicity in coastal or marine waters assesses early larval development of marine mollusks, often *Mytilus* mussels.

In traditional marine bivalve embryo-larval development tests, abnormal development is the best-recognized effect of metal toxicity at the whole-organism level (Johnson, 1988; EPA, 1995; Sussarellu et al., 2018). Abnormal development is especially apparent at 48 h post fertilization (hpf), when normal larvae reach the D-veliger stage. At this point, abnormal animals exhibit gross morphological deformities, including velum protrusions, misshapen shells, and failure to form shells (His et al., 1997; E50 Committee, 2013). This test is typically conducted as a dose response assay in which larvae are exposed to a range of concentrations and an effective concentration at which 50% of the population becomes abnormal (EC50) is determined (E50 Committee, 2013; EPA, 2016). However, the normal development assay is relatively coarse and fails to capture more nuanced and sensitive physiological responses to chemical exposure or toxicity.

Advances in “-omics” technology over the past two decades have introduced powerful tools that have vastly enhanced the sensitivity and utility of toxicity testing (Nuwaysir et al., 1999; Waters and Fostel, 2004; Calzolai et al., 2007; Schirmer et al., 2010; Hahn, 2011; Kim et al., 2015). Changes in an organism’s transcriptome or proteome in response to an introduced toxin can reveal biomarkers that are sensitive indicators of the presence of the toxin at concentrations that are below that which produce outwardly discernible effects of toxicity on the organism (Daston, 2008; Hook et al., 2014). However, to effectively harness these molecular markers, strategies are required that can classify these markers as indicators of exposure to a toxin and its presence in the environment, versus markers that indicate that the toxin is not only present but is also causing deleterious effects on the subject organism. These markers of exposure versus effect can be distinguished by phenotypic anchoring, i.e., connecting sublethal molecular changes to higher-level whole organism, population, or ecological outcomes (Tennant, 2002; Paules, 2003; Daston, 2008; Hook et al., 2014). Frameworks such as adverse outcome pathways (Ankley et al., 2010; OECD, 2013) attempt to use phenotypic anchoring to link molecular events to detrimental effects at the whole-organism level, thus identifying markers of effect (rather than exposure).

In order to identify sensitive molecular biomarkers of copper exposure, we previously investigated the concentration-responsive molecular changes associated with copper exposure in the mussel embryo-larval assay by generating expression data from pools of larvae exposed to a range of 10 copper concentrations (Hall et al., 2020). By identifying dose-responsive transcripts and comparing lowest observed transcriptional EC50 with higher level physiological outcomes (normal and abnormal development), we were able to define sensitive markers of copper response, or early warning signs that are detectable prior to the onset of morphological abnormality. Sensitive markers primarily showed repressed expression, and included genes involved in biomineralization/shell formation, metal binding, and development. Development genes were similarly down-regulated in response to low concentrations of copper in previous studies on juvenile red abalone *Haliotis rufescens*, post-larval scallops (*Argopecten purpuratus*), and early developmental stages of the oyster *Crassostrea gigas* (Zapata et al., 2009; Silva-Aciares et al., 2011; Sussarellu et al., 2018). Additionally, copper-induced down-regulation of iron and zinc binding stress-protein transcripts was observed previously in juvenile abalone (Silva-Aciares et al., 2011).

The transcriptomic analysis of Hall et al. (2020) was conducted on pooled larval samples, representative of all the larvae that were present in the culture vessel, and this pool would have included a combination of normal and abnormal larvae, the proportions of which were related to the prevailing copper concentration. While this approach has utility in relating bulk gene expression changes to copper concentration it does not address the granularity that is associated with this EC50 type of assay. The basis of this and all EC50 assays is to calculate the proportion of a test population that do or don’t exhibit some type of detrimental phenotype in response to the introduction of some toxic perturbant. Here we sought to leverage this granularity and instead of profiling a pool of all the larvae in an assay, we sought to sub-sample the larvae according to whether they exhibited a detrimental phenotype, in this case abnormal morphology, versus those that exhibited a normal phenotype. The current study advances the results of our previous efforts to identify concentration-dependent transcriptional biomarkers of copper by conducting RNAseq on phenotypically-sorted single-larvae and pools of phenotypically-sorted larvae, to distinguish markers of copper effect from those of copper exposure. Examining these subsets of normal and abnormal phenotypes provides an opportunity to understand differences in the underlying molecular pathways driving these different morphological outcomes. These higher-resolution data also corroborate previously identified markers of copper toxicity and exposure by linking individual transcriptional profiles with to larval phenotype. This work could also strengthen the adverse outcome pathway for copper toxicity in mussel larval development.

## Materials and Methods

### Broodstock Collection and Embryo Copper Exposure

Two separate experiments were run in June and September 2015 to generate the samples used in this experiment. Adult *Mytilus californianus* were collected from an intertidal site at Will Rogers State Beach, Santa Monica, CA, United States. Animals were refrigerated for approximately 6 h in preparation for spawning induced by thermal shock. Mussels were then added to a tank of filtered seawater maintained at 23°C. Once spawning commenced, individuals were removed, rinsed with 0.2 μm filtered seawater, and isolated in separate beakers containing 0.2 μm filtered seawater, collected from Big Fisherman’s Cove on Santa Catalina Island. Gametes were examined to confirm high quality, indicated for eggs by a relatively homogeneous mixture of club-shaped eggs, and for sperm by motility. After eggs transformed into a spherical shape, sperm was added to reach a density of ∼5 sperm per egg. Successful fertilization was identified by the production of a polar body. After 95% of eggs exhibited successful fertilization, embryos in the 2–4 cell stage were stocked into treatment containers at a density of ∼13 larvae/mL.

In both trials, six 1-L containers were prepared, including one control and five copper treatments (3, 6, 9, 12, and 15 μg/l). All containers were filled with 1 L of seawater (33.5 ppt) collected from Big Fisherman’s Cove on Santa Catalina Island, CA, that was 0.2 μm filtered, and heavily aerated. A 0.1 mM stock solution of copper sulfate was used to spike containers with the appropriate amount of copper. After copper addition, containers were mixed by gentle inversion. Once embryos were added to containers, they were incubated at 17°C with a 12 h D: 12 h L cycle for 48 h.

### Larval Counts and Count Analysis

At the end of the 48-h incubation period, the majority of larvae in the control had reached the D-hinge phase. The control and treatment containers were filtered through an 80 μm sieve to concentrate larvae. Larvae were then rinsed from the sieve into 50 mL Falcon tubes. The volume of each Falcon tube was recorded, and for each tube 3–5 100 μl drops were added to a Sedgewick rafter, and examined under a compound microscope. In a 100 μl drop, the average number of larvae ranged from 36 in the controls to 20 at the 15 μg/l copper concentration. Larvae used for counts were discarded after the count, and ultimately did not contribute to the sequenced larval pool. The number of normal and abnormal larvae in each drop were recorded in order to determine the proportion of survival and abnormal development. Normal and abnormal larvae were characterized in accordance with standard guidance (His et al., 1997). Normal larvae were those that had developed to the D-hinge phase and exhibited a straight hinge extending into a convex curve (shaped like a capital “D”), and abnormal larvae included normal or malformed embryos that had not yet reached the D-larval stage (typically roughly round with irregularities). Each proportion was divided by the mean control proportion 0 μg/l copper to calculate control-normalized survival and normal development. Normal development data were further analyzed in the R package ‘drc’ (Ritz et al., 2015). A four-parameter log-logistic curve (LL.4 model in the drc package) was fit to the dataset to calculate 50% normal development effective concentration (EC50) values. The survival curve was not sigmoidal, as the concentration range used in this experiment did not capture the entire scope of the survival curve. Survival was analyzed using ANOVA (r packages aov and anova). Specific differences between concentrations were further detected using a Tukey’s *post hoc* test (R command TukeyHSD).

### Sample Preservation and Sorting

Once all count samples had been taken, tubes were centrifuged at 5,000 g for 5 min; the supernatant was removed, and the remaining 1 ml of seawater containing larvae from each Falcon tube was transferred to a 2 ml tube. Approximately 500 μl of RNAlater^®^ (Ambion) was mixed thoroughly into each centrifuge tube. Samples were refrigerated overnight to allow for infiltration of RNAlater into larval tissues, and then stored at −80°C, according to the RNAlater^®^ Tissue Collection protocol.

Preserved larval samples from the control and 3, 6, and 9 μg/l copper treatments from both experiments (Trial 1- May and Trial 2 - September) were removed from the freezer and brought to room temperature. First, individual larvae were sorted using samples from the Trial 2 -September experiment. Small subsamples were removed from the tube using a Pasteur pipette, and placed in a glass dish for sorting. Because samples were highly concentrated, 1× PBS was added to facilitate visual inspection of different larval types and accurate separation. The dish was placed under a compound microscope, and 192 single larvae were isolated into PCR tubes according to whether they exhibited a normal or abnormal morphology (characteristics of normal and abnormal larvae described above) using a mouth pipetting system. Single larvae were also picked from the 9 μg/l copper treatment but these larvae were not distinguished by phenotype because 96% of larvae were abnormal at this level of copper exposure. Tubes were then re-frozen at −80°C until RNA extraction.

In addition to these isolated single-larva, normal and abnormal larvae from the Trial 1- May experiment were picked and pooled to create three replicate pools (or four pools in the case of 0-Normal samples) for each condition (0 μg/l abnormal, 0 μg/l normal, 3 μg/l abnormal, 3 μg/l normal, 6 μg/l abnormal, and 6 μg/l normal), resulting in a total of 19 pools, with about 50 animals in each pool. Photographs were taken of ∼25 larvae in each pool using a digital camera attached to a dissecting scope. The camera was set to manual focus, set at the maximum optical zoom, and fixed in this position. Similarly, the microscope was set at 40× magnification. A 1 cm stage micrometer was used to calibrate pixel to micron conversion for subsequent image analysis. Picked larval samples were then spun down quickly and excess liquid was removed. Tubes were then re-frozen at −80°C until RNA extraction.

### RNA Extraction, Library Preparation, and Sequencing

Single-larvae (Trial 2 samples) were lysed in 35 μL RLT buffer (Qiagen) containing 2 μl of silane beads (MyOne, Dynabeads) and bead-binding was induced by addition of 25 μL of ethanol. The beads were washed twice with 80% ethanol, dried for 10 min and then used as input to prepare 3′-tag RNAseq libraries using a protocol adapted from Foley et al. (2019). Briefly, the bead-bound total RNA from individual larvae was resuspended in an 8 μl reverse-transcription reaction mixture in 96-well plates with each well containing a unique indexed anchored-oligo-dT primer that contained the Illumina p7 sequence. The RNA was fragmented for 3 min at 94°C, cooled to 42°C, and then reverse transcribed by the addition of MMLV-HP reverse transcriptase (Lucigen) and a template switching oligonucleotide that contained the Illumina p5 sequence. Following reverse-transcription the plate of cDNA products was pooled, bead-cleaned (AMPure, Beckman-Coulter), and amplified for 18 cycles with Illumina p5 and p7 PCR primers. The 192 single-larvae samples were sequenced over one lane of Illumina HiSeq 4000 with 150 bp PE reads.

Pooled larval samples (Trial 1 samples) were homogenized by bead-beating, and then RNA was extracted using a modified Trizol protocol (Ambion). MaxTract columns (QIAGEN) were used to maximize phase separation and supernatant removal after chloroform addition. RNA was quantified with the Qubit HS RNA Assay Kit (Thermo Fisher), and 40 ng of each sample was used for library preparation. Prior to library preparation, each sample was combined with 4 μl of External RNA Controls Consortium (ERCC) RNA spike in mix 1 (Thermo Fisher) at a 1:10,000 dilution. Samples were poly-A selected using the NEB Next Poly(A) mRNA Magnetic Isolation Module. This step was integrated into the library preparation workflow using the NEB Next Ultra RNA Library Prep Kit for Illumina, with some modifications. Samples were fragmented for 12 min (instead of 15) prior to cDNA synthesis, and the first strand synthesis reaction was run for 50 min at 42°C. PCR enrichment was visualized using a Bio-Rad qPCR Thermocycler, and the reaction was terminated shortly after entering the exponential amplification stage. PCR amplification of libraries was run for 18 cycles. Library sizes and quantity were analyzed on a Bioanalyzer, and quantity was additionally measured with qubit. Samples were pooled and sequenced over one lane of Illumina HiSeq 4000 with 50 bp SR reads.

### Assembly and Annotation of *de novo* Transcriptome

Three *M. californianus* libraries were integrated to generate a *de novo* transcriptome assembly, as described in Hall et al. (2020), with the following modifications.

Prior to assembly, common contaminating sequences were filtered from the two Illumina libraries using bbmap.sh by mapping SE reads, merged PE reads, and unmerged PE reads to the DH10B E. coli genome and the NCBI UniVec database (minid = 0.85, idfilter = 0.90). The Sanger assembly was also filtered using BLAST (blastn, perc_identity = 90), and only contigs with an alignment length greater than 100 bp with a contaminant database target were removed.

Illumina libraries were mapped to the Sanger assembly with bbmap.sh (minid = 0.85, idfilter = 0.90), and unmapped reads were written as output. Unmapped reads were assembled using idba_tran (IDBA v1.1.1, –merge –filter, Peng et al., 2013) with maxk = 124. The two resulting assemblies were combined with the Sanger assembly, and redundant sequences were merged with CD-HIT-est v4.6.5, with c = 0.95 (Li and Godzik, 2006; Fu et al., 2012), two rounds of CAP3 [o = 50, *p* = 98, (Huang and Madan, 1999)], one round of minimus2 [OVERLAP = 50, MINID = 98, (Treangen et al., 2011)], and one final round of CD-HIT-est. Mitochondrial sequences were filtered from the assembly by running blastn (Altschul et al., 1990) against the *M. californianus* mitochondrial genome (perc_identity = 90, alignment length ≥ 100), and contigs shorter than 200 bp were removed using seqmagick (convert, min-length = 200, Matsen Group, 2017).

In addition to the annotation pipeline described in Hall et al. (2020), annotations were retrieved using blastn (outfmt ‘6 std stitle staxids’) against the NCBI EST and nt databases, and diamond blastx and blastp [taxonmap ∼/prot.accession2taxid.gz –taxonnodes ∼/nodes.dmp –more-sensitive –outfmt 102 –max-target-seqs s10 –evalue 1e-5; (Buchfink et al., 2015)] against the NCBI nr database. The full taxonomic path was retrieved for BLAST and diamond BLAST output by joining taxon IDs with a parsed file that joined taxon ID and taxonomic path (R. Sachdeva, pers. comm. 2017). All taxonomy annotations were combined into one file, and the number of metazoan annotations per contig were counted. Contigs that were metazoan for all of the annotations were kept. Additionally, predicted peptides with metazoan taxonomy in blastp results against UniProt and nt were kept. Finally, contigs that annotated as metazoan for all BLAST searches, but could not be resolved below “root,” “cellular organism,” “Eukaryota,” or “Opisthokonta” for diamond blast taxonomy searches, were kept as well. The final assembly consisted of 71,451 contigs with an average length of 1142.73 bp.

### Downstream Data Analysis

The following process was run separately for sorted pooled larval samples (Trial 1) and single larval samples (Trial 2). Raw RNAseq reads were quality trimmed and contaminating adapter sequence was removed using Trimmomatic v0.33 (Bolger et al., 2014) with default parameter settings. The trimmed reads were then mapped to the *M. californianus* mitochondrial genome using BBMap v34 (minid = 0.95 ambiguous = all sssr = 1.0) (Bushnell, 2016) to separate mitochondrial transcripts from nuclear genes. All reads that did not map to the mitochondrial genome were used for subsequent analysis. Larval reads were mapped to the *de novo* transcriptome assembly described above with bbmap.sh (minid = 0.95 for pooled larvae, default for single larvae, ambiguous = random, sssr = 1.0, nhtag = t, minlength = 40). The resulting bam files were counted and summarized with featureCounts (Liao et al., 2014), allowing for multimapping reads (-M), and allowing for mapped reads overlapping two contigs to be counted toward those contigs (-O).

Count tables were loaded into R (R Core Team, 2016) and processed in DESeq2 (Love et al., 2014). Initial inspection of the PCA plot of normalized transcriptional counts for pooled larvae revealed that there were two outliers, one replicate of normal animals at 0 μg/l copper, and one normal animal replicate at 3 μg/l copper. These two samples also proved to be outliers in a PCA of only the ERCC reads, which one would expect to be relatively consistent across samples after normalization. Therefore, these samples were removed from downstream analysis. For the remaining 17 samples, reads with counts higher than 40 were removed in the initial filtration. Inspection of the PCA plot of 192 normalized transcriptomes for single larvae revealed several outliers, which were confirmed and supplemented by examining a boxplot of the Cook’s distance for all single larval samples. Both of these approaches revealed 6 outlier samples which were removed from downstream analysis. All subsequent analysis was performed on the remaining 186 samples, which comprised 48 control larvae, and 46, 70, and 22 larvae sampled at 3, 6, and 9 μg/l copper, respectively.

DESeq2 was used to further process both datasets, according to the standard workflow, and significant differentially expressed (DE) genes were detected between group pairs. The entire process was run twice with different grouping assignments—the first, which was used to identify markers of exposure, grouped all 0 μg/l, all 3 μg/l, and all 6 μg/l copper-treated larval samples (as opposed to grouping by morphology in addition to copper), and compared 0 μg/l with 3 μg/l, and 0 μg/l with 6 μg/l. The second grouping assignment used factors that distinguished samples by both copper concentration and morphology, and compared normal and abnormal animals at 0, 3, and 6 μg/l. DE genes identified by each of these approaches were further filtered to retain only those that demonstrated significant changes in expression (*p*_*adj*_ < 0.1, according to the DESeq2 protocol), and a fold-change > 2.3.

**FIGURE 1:**
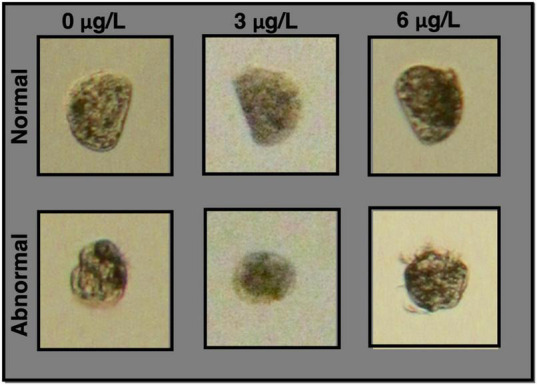
Images of normal and abnormal larvae at each copper concentration. Normal animals are morphologically very similar for all copper concentrations, and were characterized by standard features of larvae that have reached the D-veliger stage – a straight hinge on one side of the organism, and a regular convex curve extending out from the hinge. More variation was observed in abnormal larvae, but key defining features were round and/or irregular morphology.

**FIGURE 2:**
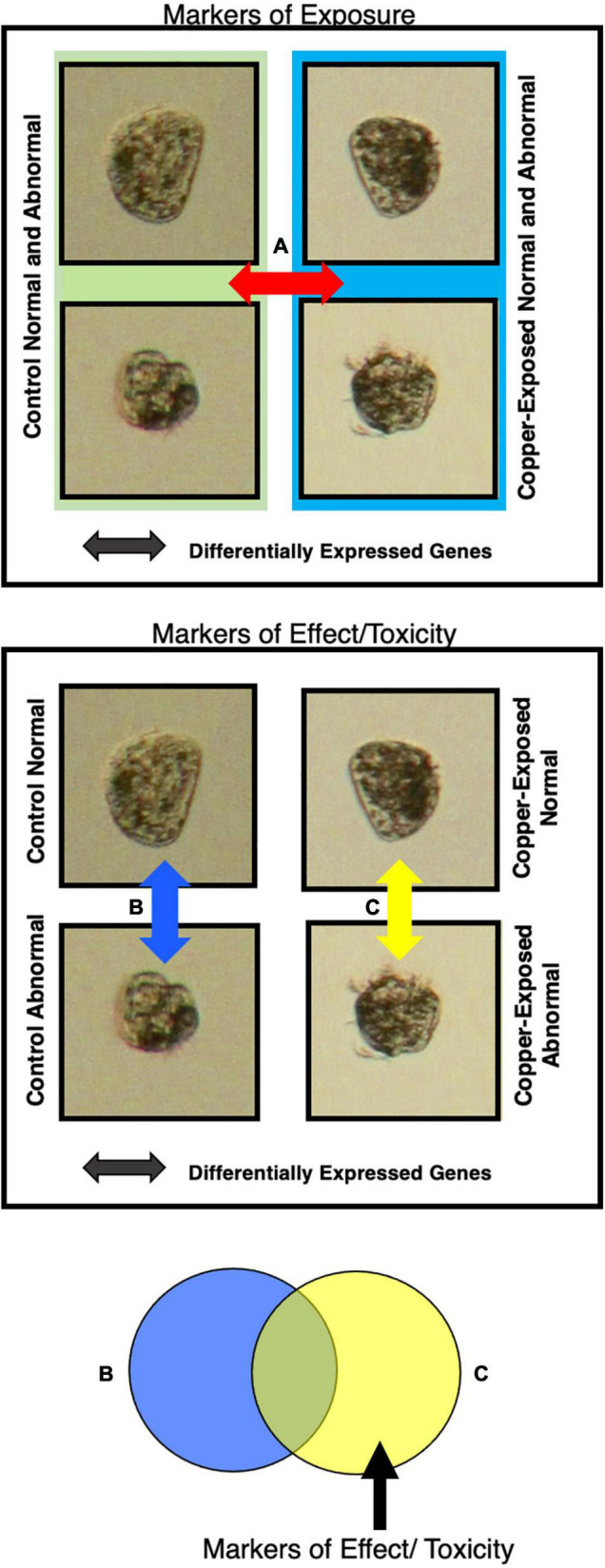
Markers of effect and markers of exposure were detected by isolating gene sets that were differentially expressed between animals exposed to different copper concentrations and that exhibited different morphologies. Markers of exposure were considered genes that were differentially expressed between all animals (normal and abnormal) at the control copper concentration and all animals at each copper concentration **(A)**. Markers of effect were considered genes that were differentially expressed between normal and abnormal animals in copper-treated larval samples, but not in control samples **(B,C)**.

To explore the genes driving the observed differences in morphology (Figure 1), differential expression (DE) was assessed between conditions. Specifically, we identified markers of copper exposure and markers of copper toxicity by extracting unique and overlapping groups of DE genes (Figure 2). Markers of copper exposure were defined as genes that were DE between all control animals (0 μg/l) and animals at both copper concentrations (3 and 6 μg/l), as exposure markers should be evident in all animals exposed to a toxin. Markers of toxicity were defined as genes that were DE between normal and abnormal animals at 3 μg/l copper, 6 μg/l copper, or at both copper concentrations (Figure 2). Abnormal development is the detrimental phenotype that was used to anchor markers of effect/toxicity. Markers of natural abnormality (as opposed to copper-induced abnormality) were excluded from the analysis by excluding genes DE between normal and abnormal animals at 0 μg/l copper.

Comparison of markers of exposure lists and markers of effect lists generated for the two datasets – pooled and single larval – was conducted in R. Both datasets were searched for overlapping biomarkers and biomarkers of interest from past studies.

### Functional Analysis

Functional enrichment analysis was conducted using Gene Ontology (GO) (Ashburner et al., 2000) terms using the Cytoscape (Shannon et al., 2003) plug-in, BiNGO (Maere et al., 2005). Overrepresentation was tested using a hypergeometric test with Benjamini & Hochberg FDR correction (*p* < 0.05). The GO annotation file was generated using GO annotations produced by Trinotate, and only annotations for the 27,642 filtered contigs were included. The most recent core ontology file (go.obo) was used for analysis^1^ (October 2017).

### Figures

All figures were generated in R studio (version 3.3.1—RStudio Team, 2017). Survival was plotted with ggplot2 (Wickham, 2009); normal development was plotted using the drc function plot.drm; and venn diagrams were plotted with the package VennDiagram (Chen, 2017). PCA plots were generated in DESeq2, and heatmaps were created using the pheatmap package (Kolde, 2015). Transformed counts for heatmaps and PCA plots were calculated with Variance Stabilizing Transformation, using the DESeq2 function vst. This method is recommended for normalizing data for visualization according to the DESeq2 protocol. Average counts were taken for replicates, and averages were divided by control counts so the control count would be 1 for all samples.

## Results

### Survival and Normal Development

**FIGURE 3:**
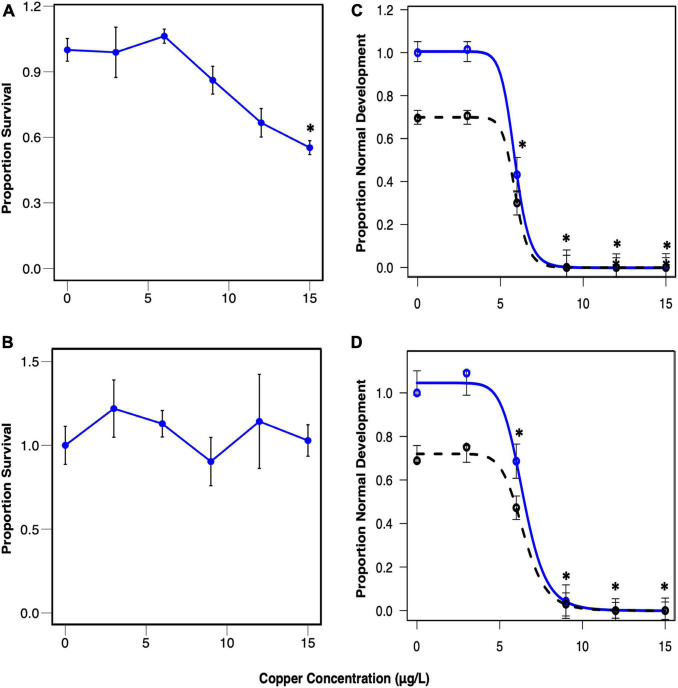
Proportion of control-normalized survival in Trial 1 **(A)** and Trial 2 **(B)** and normal development in Trial 1 **(C)** and Trial 2 **(D)** plotted against copper concentration. Mean survival with standard error **(A,B)** and mean normal development with standard error and modeled 4-parameter log-logistic curves **(C,D)** are plotted. Blue points and lines represent control-normalized survival **(A,B)** and normal development **(C,D)**, while the black dashed line represents non-normalized normal development. Asterisks indicate concentrations that exhibited significantly different proportions from the control (*p* < 0.005). The normal development EC50 was 5.87 μg/L for the pooled larvae trial (Trial 1), and 6.43 μg/L for the single larvae trial (Trial 2).

Larval survival and normal development for both experiments are depicted in Figure 3. Survival rates were relatively high across all concentrations in these experiments, so the concentration range did not capture the full survival response curve (Figures 3A,B), and it was not possible to calculate the LC50. Slight hormesis was observed at 3 and 6 μg/l copper in Trial 1, and 3, 6, and 9 μg/l copper in Trial 2, resulting in higher survival rates at these concentrations. Normal development in the control was on average 69% of the total population in both trials (Figures 3C,D). Normal development exhibited a classic sigmoidal dose response curve (Figures 3C,D), and the EC50 was 5.87 and 6.43 μg/l in Trials 1 and 2, respectively.

### Transcriptional Patterns and Morphology

**FIGURE 4:**
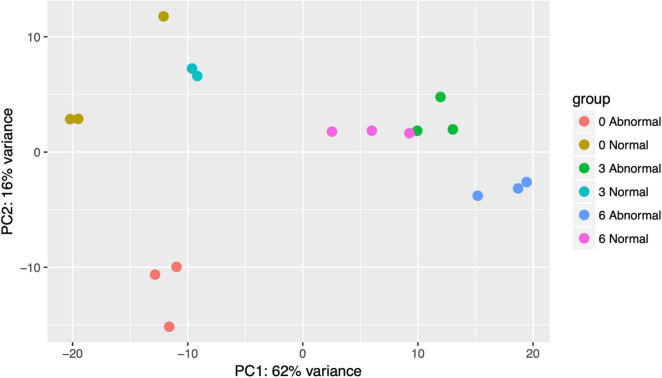
A PCA plot was created of pooled filtered larval transcriptomes (total gene count >40 across all samples). Point colors are unique to copper concentrations and morphologies. Counts were normalized in DESeq2 and transformed with variance stabilizing transformation (vst) prior to plotting.

**FIGURE 5:**
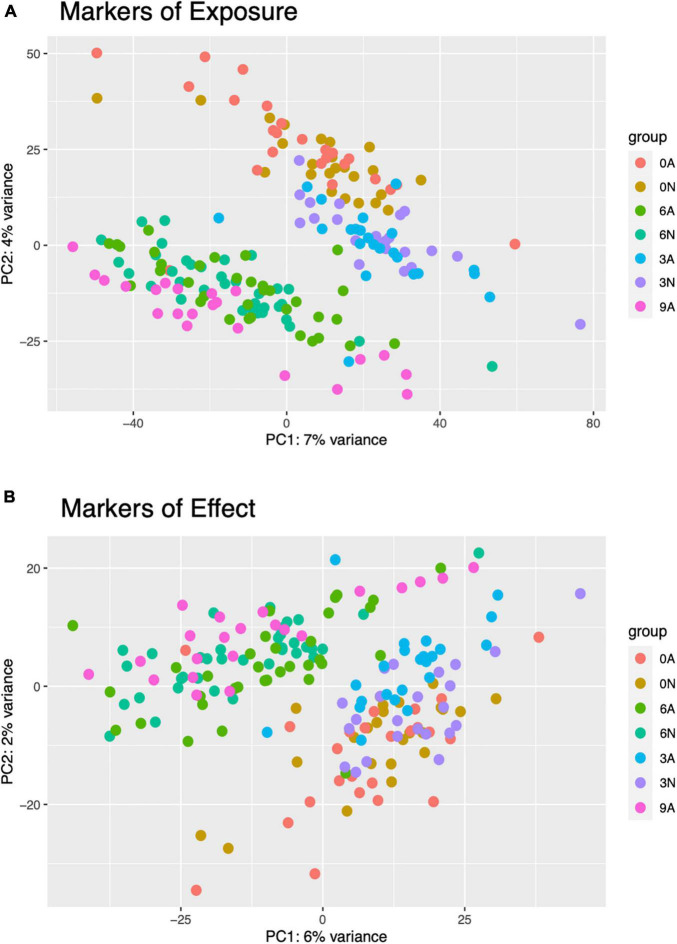
PCA plots were created for single larval markers of exposure **(A)** and effect **(B)**. Point colors are unique to combined copper concentration (0, 3, 6, or 9 μg/L) and morphologies (N, normal, or A, abnormal). Counts were normalized in DESeq2 and transformed with variance stabilizing transformation (vst) prior to plotting.

**FIGURE 6:**
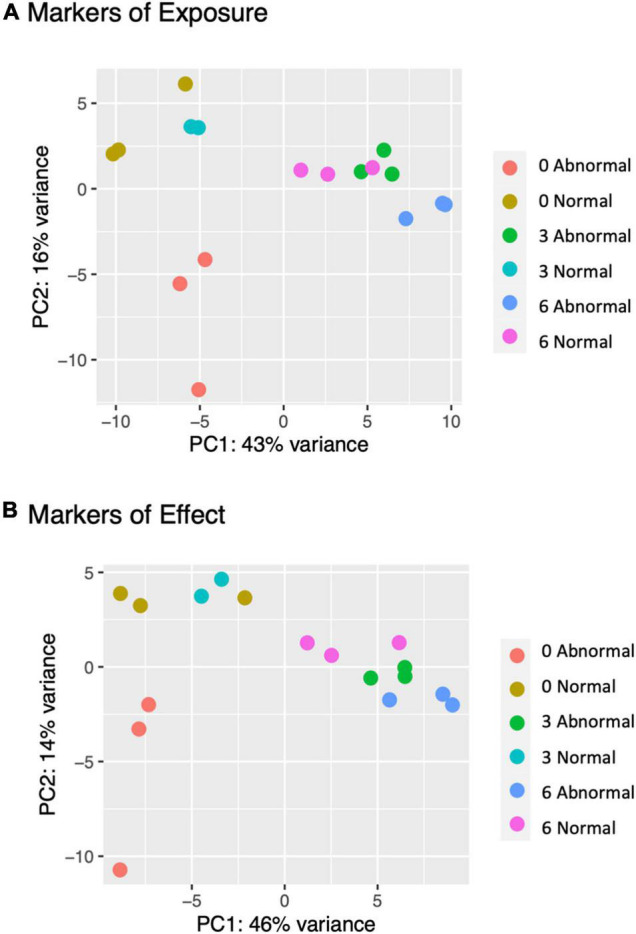
To corroborate trends observed in individual larvae, pooled larval expression data was subset on the markers of exposure and effect generated through single larval analysis. PCA plots of this expression data for markers of exposure **(A)** and effect **(B)** confirmed that single larval markers effectively separated pooled larval samples based on morphology and copper concentration.

Principal Component Analysis (PCA) of pooled larval transcriptional profiles revealed that replicate samples clustered by copper concentration and morphological condition (Figure 4). Three broad clusters of samples were apparent. The first cluster consisted solely of the samples of abnormal animals cultured under control conditions (0 μg/l copper), indicating that larvae that exhibited abnormal development under control culture conditions possess a different gene expression signature to those that exhibit abnormal morphology under copper exposure. The second cluster represented a grouping of samples of normal animals from the control (0 μg/l copper) and the 3 μg/l copper treatments, while the third cluster comprised samples from abnormal animals from the 3 μg/l copper treatment, and both the normal and abnormal animals exposed to 6 μg/l copper. A PCA of whole single larval transcriptional profiles revealed a clear gradient in sample concentration, but did not distinguish between normal and abnormal samples. When filtered to focus on markers of exposure and effect, however, single larval samples did separate by low (0 and 3 μg/l) and high (6 and 9 μg/l) copper concentrations (Figure 5), and in the markers of effect samples could be distinguished by morphology in the 3 μg/l copper concentration (Figure 5B). Furthermore, when expression of genes that were identified as markers of exposure and effect in single larval samples were projected using PCA on the pooled larval dataset, the same pattern apparent in the pooled larval markers of exposure and effect was apparent – samples separated based on morphology at 0 and 3 μg/l copper, but not at 6 μg/l copper (Figure 6). Thus, patterns of gene expression observed in data collected at single-larva resolution was recapitulated in an independent dataset collected using pooled larvae and showed that gene expression was able to robustly distinguish larvae based on morphology at 3 μg/l copper, but that such transcriptional signatures were dampened at 6 μg/l.

### Markers of Exposure

**FIGURE 7:**
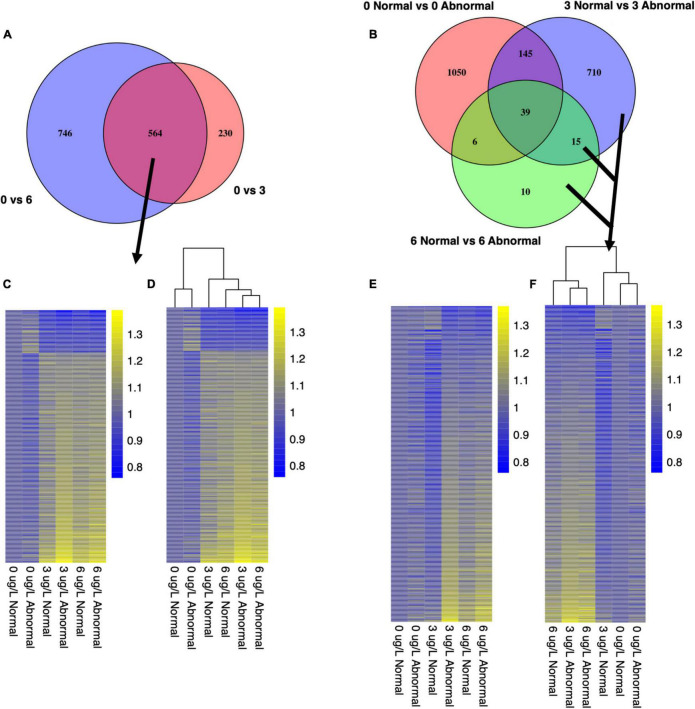
Venn diagrams illustrate gene sets that were chosen as pooled larval markers of exposure **(A)** and markers of effect **(B)**. Heatmaps depict expression patterns of shared markers of exposure **(C,D)** and all markers of effect **(E,F)**. Counts were transformed using Variance Stabilizing Transformation in DESeq2. Each column represents the control-normalized mean count for all replicates in a given condition. Yellow coloration represents higher expression values, and blue coloration represents lower expression values.

For pooled larval samples, 564 genes were differentially expressed between all control animals and all copper-exposed animals at both concentrations (Figure 7 and Supplementary Table 1). A total of 230 additional genes were only DE between control and 3 μg/l samples, yet 746 genes were uniquely expressed between control and 6 μg/l samples (Figure 7). Of the common set of 564 DE genes, 469 were upregulated in expression relative to the control copper condition, and 95 were downregulated in expression relative to the control copper condition (Figures 7C,D and Supplementary Table 1). For single larval samples, 1,242 genes were differentially expressed between all control and all copper-exposed animals at 3 and 6 μg/L. There were an additional 2,595 genes that were only DE between control and 3 μg/L samples, and 3,718 DE genes between control and 6 μg/L samples.

**FIGURE 8:**
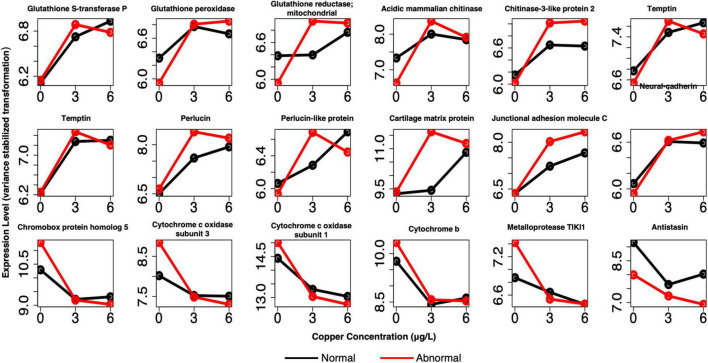
Example profiles of pooled markers of exposure. Genes are related to oxidative stress, shell formation, cell adhesion, and other processes. Red lines depict expression of abnormal animals, and black lines depict expression of normal animals.

In pooled larvae, many of the identified markers of exposure were related to cell adhesion, extracellular proteinaceous matrix, and shell formation (Figure 8 and Supplementary Table 1). We identified several shell formation markers that have appeared in previous larval investigations, including temptin, perlucin, and chitin-related genes (Hall et al., 2020). Additional markers related to proteinaceous matrix, adhesion, and shell formation were identified, including insoluble matrix shell protein 5, matrix metalloproteinase-16, junctional adhesion molecule C, periostin (*POSTN*), neural-cadherin, and a disintegrin and metalloproteinase with thrombospondin motifs 13. Other markers included several well-recognized markers of oxidative stress, including glutathione-s-transferase P (*GSTP1*), mitochondrial glutathione reductase (*GSR*), and glutathione peroxidase (*GPx*), as well as putative DBH-like monooxygenase protein 2, which has oxidoreductase activity. All of these markers were upregulated relative to the control in copper conditions. Downregulated markers of exposure did not exhibit any specific trends in functional category, and included genes such as chromobox protein homolog 5, cytochrome c oxidase subunits 1 and 3, cytochrome b, metalloprotease TIK12, amine sulfotransferase, and antistasin. Many of these same markers were identified in single larval samples as well (Supplementary Table 2), although markers related to shell formation and oxidative stress/xenobiotic protection were present in greater numbers in the markers of effect.

The GO terms enriched in these common biomarkers of exposure in the pooled larval samples were primarily related to the same processes described above. There were two chitin-related terms: chitin binding and chitin metabolic process (Supplementary Table 3). Several terms were involved in development, including neuron projection extension, and negative regulation of cell development; while there were also terms related to healing and tissue regeneration. Finally, several terms were related to peptidase/hydrolase activity and regulation, as well as chemokine and cytokine secretion. In the single larval markers of exposure, only two GO terms were enriched, both related to non-membrane bound organelle.

### Markers of Effect

To identify markers of effect, we investigated transcriptional markers associated with abnormal development in low to mid-range copper concentrations (Figure 1). In these treatments, some organisms exhibited normal development at the end of 48 h, while others became abnormal, despite exposure to identical conditions of copper exposure. Markers of effect (or copper-induced abnormal development) were identified as the set of genes that were DE between normal and abnormal larvae at both 3 and 6 μg/l (Figure 2). Because larval abnormality also occurs in the absence of copper, we first identified 1,240 genes as DE between normal and abnormal animals at 0 μg/l copper in pooled larval samples (Figure 7B), and 2,358 genes DE between normal and abnormal animals at 0 μg/l for single larval samples. These genes represent transcriptional markers of spontaneous natural abnormality under control conditions and therefore we excluded these genes from further consideration as candidates markers of copper exposure and effect. After subtracting the genes that were associated with natural abnormality under control conditions, there were 735 genes that appeared to be markers of copper induced abnormality in pooled larvae, and 2,792 markers of copper induced abnormality in single larvae. The number of DE genes between copper-exposed normal and abnormal animals was 909 at 3 μg/l copper, and 70 at 6 μg/l copper for pooled samples. For single larval samples 1,848 genes were DE between copper-exposed and abnormal animals at 3 μg/l copper, and 1,805 at 6 μg/l. There were 163 shared markers of effect at both copper concentrations, but 1,267 markers of effect were unique to 3 μg/l, and 1,370 markers were unique to 6 μg/l.

In pooled larval samples, abnormal phenotypes were generally associated with induction of transcripts relative to normal phenotypes, with 90% of transcripts more highly expressed in abnormal animals at 3 μg/l, and 76% expressed more highly in abnormal animals at 6 μg/l (Figures 7E,F and Supplementary Table 4). In single larval samples at 3 μg/l, this same trend was observed, although not as strongly, with 53% of transcripts more highly expressed in abnormal animals. However, at 6 μg/l, the majority of markers (59%) were expressed more highly in normal larvae.

**FIGURE 9:**
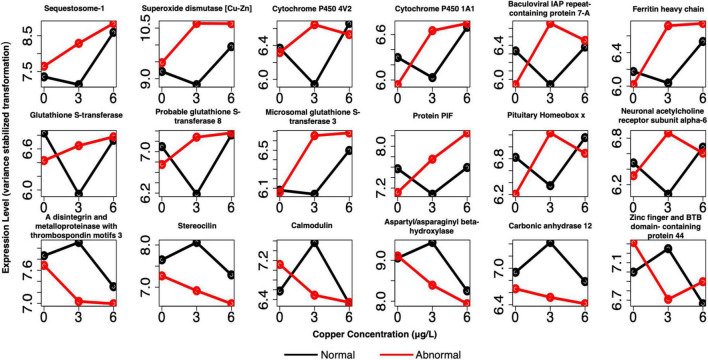
Example profiles of pooled markers of effects at 3 μg/L copper. Genes are related to apoptosis, oxidative stress, shell formation, development, cell adhesion, and divalent cation binding. Red lines depict expression of abnormal animals, and black lines depict expression of normal animals.

For pooled larval samples, many notable genes were DE between normal and abnormal animals at 3 μg/l copper (Figure 9 and Supplementary Table 4). Prominent categories that were evident in this group were similar to those that appeared in the markers of exposure. However, more representative genes were often present among markers of effect in these shared categories relative to the markers of exposure, especially among the single larval markers (Supplementary Table 5). Genes related to oxidative stress and redox cycling were again evident, including several glutathione-*s*-transferases, putative ferric-chelate reductase 1 homolog, peroxidasin, peroxidase-like protein, superoxide dismutase [Cu-Zn] (*SOD1*), several cytochrome P450 subunits, and ferric chelate reductase 1. Several protein matrix/shell formation genes appeared again as well, including matrix metalloproteinase-17, protein PIF (*pif*), peroxidasin, and carbonic anhydrase 12. Genes involved in apoptosis were also more highly expressed in abnormal animals at 3 μg/l and included baculoviral IAP repeat-containing protein 7-A (*birc7-a*), ferritin heavy chain (*FTH*), and sequestosome-1 (*Sqstm-1*).

Other markers were involved in development and neuron function, including sodium/potassium/calcium exchanger 4, neuronal acetylcholine receptor subunits alpha-3, alpha-10, and alpha-6; pituitary homeobox x, homeobox protein extradenticle, and membrane metallo-endopeptidase-like 1 (Figure 9 and Supplementary Table 4). Finally, several unique genes related to cell adhesion belonged to this set as well. These genes were protocadherin-16, a disintegrin and metalloproteinase with thrombospondin motifs 16, and a disintegrin and metalloproteinase with thrombospondin motifs 3 (*ADAMTS3*). Many of these markers, or markers with very similar function, were again identified as markers of effect in the single larval samples (Supplementary Table 5). They include a number of glutathione-s-transferases, glutathione peroxidase, peroxidasin, putative ferric-chelate reductase 1 homolog, several cytochrome p450 subunits, *pif*, perlucin (also a shell formation gene), a number of hox genes, and *ADAMTS16*.

The above genes were upregulated in abnormal animals in pooled larval samples, and primarily upregulated in single larval samples, although several were downregulated in abnormal animals in single larvae. Genes that were downregulated in abnormal animals in pooled larval samples also included several cell adhesion genes (*ADAMTS3* and stereocilin), as well as calcium and zinc binding genes (calmodulin, aspartyl/asparaginyl beta-hydroxylase, carbonic anhydrase 12, zinc finger and BTB domain-containing protein 44, MORC family CW-type zinc finger protein 2A, synaptotagmin-like protein 5, and PHD finger protein 14). Again, no notable trends were apparent among downregulated genes.

Five GO terms were enriched in the markers of effects at 3 μg/l copper: for pooled larvae chitin binding, chitin metabolic process, amino sugar metabolic process, glucosamine-containing compound metabolic process, and extracellular region (Supplementary Table 6). Many more GO terms were enriched in the single larval markers of effect. Enriched GO terms were related to RNA/mRNA splicing, RNA binding, non-membrane bound organelles, cytoskeleton, RNA localization, regulation of cell cycle process, and nuclear lumen (Supplementary Table 7).

In addition to the discrete biological replicates that were sorted and sequenced in this experiment through the pooled and single larval sequencing, we can rely on data from a recent publication, Hall et al. (2020), in which similar concentration-response experiments were conducted with *M. californianus* larvae, as a repeat for this study. In Hall et al. (2020), we conducted two concentration response experiments in which two families of *M. californianus* larvae were exposed to 10 copper concentrations, and whole sample transcriptomes were sequenced. The EC50 for this experiment was similar to the other two biological replicates in the aforementioned study, and transcriptional markers identified in this manuscript are likewise similar to the transcriptional markers identified in the previous study. A comparison of the markers of exposure and effect identified in this study against markers that were identified as showing a significant dose response profile in our previous study shows that 55% of the markers of exposure, and 64% of the markers of effect were previously identified as copper-responsive. Additionally, we examined the expression profiles of the identified markers of exposure and effect in the dataset of Hall, Moffett, and Gracey (Supplementary Figure 1). The heatmaps in Supplementary Figure 1 confirm that the majority of these markers exhibited a transcriptional response to copper in our previous study, demonstrating that these genes are consistently differentially expressed to copper across experiments.

### Amplitude-Dependent Markers of Exposure and Effect

**FIGURE 10:**
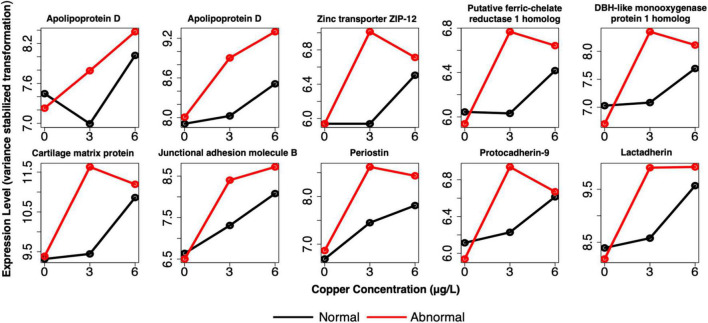
Example expression profiles in pooled larvae of a subset of the genes that were identified as both markers of exposure and effect. Genes are related to apoptosis, oxidative stress, shell formation, development, cell adhesion, and divalent cation binding. Red lines depict expression of abnormal animals, and black lines depict expression of normal animals.

Comparison of the biomarkers of effect at 3 μg/l with biomarkers of exposure revealed that 59 genes were shared between the two gene sets (Supplementary Table 8). These markers predominantly consisted of genes that are DE in copper-exposed larvae, but whose expression was amplified in abnormal larvae. The expression of 97% of genes was amplified in abnormal larvae, whereas expression was reduced for only 3% of genes (Figure 10 and Supplementary Table 8). The amplitude-dependent markers were related to oxidative stress and/or oxidoreductase activity (e.g., apolipoprotein D, putative ferric chelate reductase 1 homolog, cytochrome P450 subunits, and DBH-like monooxygenase protein 1 homolog); extracellular/proteinaceous matrix formation (putative tyrosinase-like protein tyr-3, and cartilage matrix protein); and cell adhesion [junctional adhesion molecule B (*JAM2*), *POSTN*, protocadherin-9 (*PCDH9*), and lactadherin]. For several additional genes related to cell adhesion, two separate copies of the gene appeared in each set of markers, respectively. These genes included integrin beta-5; cadherin 99C; and protocadherin Fat 1. Two other notable genes that were identified as amplitude-dependent markers were zinc transporter ZIP12, and serine/threonine-protein phosphatase 2A, both of which bind divalent metals. For single larvae, 228 genes were shared markers of exposure and effect, but these genes did not consistently exhibit amplified expression in abnormal larvae. For this gene set, markers were both upregulated and downregulated in response to copper, and both upregulated and downregulated in abnormal larvae relative to normal larvae. The directionality of response was not consistent for markers of exposure and effect (i.e., upregulation in all copper-exposed larvae was sometimes associated with higher expression in normal larvae, rather htan normal larvae).

### Markers of Natural Abnormal Development

Beyond markers of copper exposure or effects, we also identified markers of natural spontaneous abnormality as depicted in Figure 2B. In pooled larval samples, 1,240 genes were DE between normal and abnormal animals, and of these 380 genes were up-regulated in abnormal larvae relative to normal larvae, and 860 genes were down-regulated in abnormal larvae relative to normal larvae. In single larval samples, 2,358 genes were DE between normal and abnormal animals, and of these 1,600 were up-regulated in abnormal larvae relative to normal larvae, and 758 were down-regulated in abnormal larvae relative to normal larvae. Prominent functions of genes identified among the DE genes include development, extracellular matrix, cytoskeletal components and motility, cell cycle, shell formation, transmembrane proteins, protease inhibitors, oxidative stress/protein turnover, neurotransmitters, and replication/transcription (Supplementary Tables 9, 10). In the pooled markers of natural abnormal development, there were also several groups of similar genes that appeared in the DEG list – 5 GTP binding proteins, 4 heat shock proteins, 5 hemicentins, 6 serine/threonine-protein kinase or phosphatases, 8 solute carrier family members, 5 WD repeat-containing proteins, and 5 zinc finger proteins. Although many of the functional groups represented by this gene set were also common in DE genes in copper-exposed abnormal animals, genes were unique to this gene set, as they were removed from the markers of effect, indicating that there are many distinct markers of natural abnormality and copper-induced abnormality.

## Discussion

Phenotypic anchoring of transcriptional biomarkers is a common and necessary approach to ultimately distinguish biomarkers of exposure from those of effect (Paules, 2003; Daston, 2008; Hook et al., 2014). In this study, we used larval morphology to anchor gene expression profiles. The normal development EC50s of 5.87 and 6.43 μg/l copper agreed with previous work on *Mytilus* larvae (Martin et al., 1981; Arnold et al., 2009; Hall et al., 2020), indicating that expression results from this culture are suitable for extrapolation to other studies.

Generally, normal and abnormal larvae in pooled samples exhibited distinct, phenotype-dependent transcriptional responses (Figure 2), as we would expect, which was important for parsing out markers of exposure and effect. However, the transcriptional similarity between normal and abnormal animals at 6 μg/l was somewhat surprising. The fact that transcriptional profiles are significantly different for normal and abnormal animals at 0 and 3 μg/l copper, but not at 6 μg/l, suggests that as copper concentrations increase, the transcriptional signature of toxicity becomes the dominant expression signature, even in morphologically normal animals. While morphology-based transcriptional differences weren’t immediately apparent in the single larval data, large numbers of genes were differentially expressed between normal and abnormal larvae at each copper concentration, indicating that there were in fact notable morphology-linked expression patterns. Distinct expression patterns based on morphology were apparent in the PCA-plotted markers of effect at 3 μg/l, but not at 6 μg/l (Figure 5B). Thus, in both pooled and single larvae, markers of effect appear to be the most useful at low copper concentrations, but many markers of effect were still evident at the mid-range copper concentration (6 μg/l) when single larval sequencing was used.

While we identified unique markers of exposure and effect, clearly indicating that these do comprise two distinct gene sets, markers of exposure and effect were involved in many similar functional pathways. Biomarkers of copper exposure and effects were related to oxidative stress or redox reactions, cell adhesion, and shell formation/extracellular proteinaceous matrix, which is consistent with our previous analysis of mussel larval response to copper (Hall et al., 2020), and shares some similarities with other previous studies on marine larval response to copper (Zapata et al., 2009; Silva-Aciares et al., 2011; Sussarellu et al., 2018). The pathways identified provide insight into the possible mechanisms of copper-induced abnormal development in mussel larvae.

Several genes related to oxidative stress or oxidoreductase activity were uniquely identified as markers of effect, and not markers of exposure (Figure 9 and Supplementary Table 4). In the pooled larval samples, *SOD1* and *FTH* were identified as unique markers of exposure. *SOD1* uses copper ions to oxidize superoxide molecules (Valentine and Mota de Freitas, 1985) and is a well-known component of the oxidative stress response (Finkel and Holbrook, 2000). *FTH*, a marker of abnormal development at 3 μg/l copper, plays a role in sequestering and oxidizing excess ferrous ions to prevent oxidative stress (Orino et al., 2001). In both pooled larvae and single larval samples, glutathione-related markers appeared in the markers of exposure and effect (Figures 8, 9 and Supplementary Tables 1, 2, 4, 5), but unique Glutathione *S*-transferases were identified as markers of effect. In single larval samples, Glutathione *S*-transferases only appeared as markers of effect. Glutathione *S*-transferases are known to play distinct roles in the oxidative stress response (Veal et al., 2002) and in xenobiotic detoxification in general (Salinas and Wong, 1999), as is glutathione peroxidase (Freedman et al., 1989). Several cytochrome P450 subunits were identified as unique markers of effect as well. Cytochrome P450s are iron-bound monooxygenases that have been implicated in the generation of reactive oxygen species (Lewis, 2002).

Previous transcriptional studies exposing marine mollusk larvae to copper have confirmed that similar genes are involved in redox regulation or protection against oxidative stress, including glutathione-s transferases, cytochrome P450 subunits (Hall et al., 2020), glutathione peroxidase, and ferritin (Zapata et al., 2009). The finding of oxidative stress in copper-exposed early bivalve larvae is further validated by Sussarellu et al. (2018), who observed genotoxicity, measured by DNA breaks, in larval oysters exposed to low copper concentrations. The modulation of distinct oxidative stress genes in both markers of exposure and markers of effect indicates that both normal and abnormal animals experience oxidative stress, as we would expect, but exercise unique physiological responses, which may be a contributing factor to their ultimate morphological state (e.g., perhaps the pathways activated in normal animals more effectively dampen oxidative stress, and thus reduce cellular damage that could lead to abnormal development). Alternatively, activation of these genes could be indicative of additional detoxification necessary in abnormal animals, but not in all copper-exposed animals. In this scenario, it is possible that standard cellular processes that would regulate redox activity and mitigate the production of free radicals are disrupted as a function of abnormal development, and therefore these animals need to scale up defenses against oxidative stress. This is supported by the genes involved in oxidative stress or redox cycling in the amplitude-dependent markers of exposure (Supplementary Table 8 and Figure 10), which suggest that the oxidative stress response is more strongly induced in markers of effect, and that higher expression levels of these genes in abnormal animals can be considered markers of effect at 3 μg/l copper.

Several previously identified indicators of damaged protein turnover and cellular damage appeared in the markers of effect and exposure (Figure 9 and Supplementary Tables 2, 4, 5). Sqstm1, which codes for a zinc-binding protein involved in protein degradation (Seibenhener et al., 2004), appeared in the markers of effect in pooled larvae, and markers of exposure in single larvae. Sqstm1 is a robust biomarker of copper exposure and is highly induced in response to copper and is consistently highly expressed in both larval and adult mussels exposed to copper (Hall et al., 2020). *Birc7-a* likewise codes for a zinc-binding protein, and it is essential to the regulation of apoptosis and cell proliferation. This gene was a marker of effect in both pooled and single larvae.

Genes related to larval shell proteinaceous matrix were present in both markers of exposure and effect, and in single larval samples they were notably more prominent in the markers of effect (Figures 6–8 and Supplementary Tables 2, 4). Many genes were related to processing of chitin, which is known to be a core component of the molluscan shell proteinaceous matrix (Weiner et al., 1984; Furuhashi et al., 2009), and has specifically been demonstrated to perform an important role in formation and function of early larval *Mytilus galloprovincialis* shells (Weiss and Schönitzer, 2006). Chitin binding and chitin metabolic process GO terms were enriched in markers of exposure and low concentration markers of effect in pooled larvae. The markers of exposure included chitinase 3-like protein 2, acidic mammalian chitinase, collagen alpha-1(XII) chain, and lactase-phlorizin hydrolase, and the markers of effect included chitotriosidase-1, collagen alpha-4(VI) chain, *pif*, inactive carboxypeptidase-like protein X2, and beta-hexosaminidase. Chitin-related genes also responded to copper at relatively low concentrations in our previous study and have thus consistently represented good early markers of copper effects (Hall et al., 2020). Considering the clear impacts of copper on mussel larval development and shell formation, and the integral role that chitin plays in larval shell formation, it makes sense that this group of genes were identified in the copper response. Modulation of chitin-related genes in abnormal animals could be a compensation mechanism to address the damaged shell matrix associated with abnormal development. Chitin-related genes have also been identified as markers of zinc exposure in Daphnia magna (Poynton et al., 2007), and of copper exposure in adult mussels (Negri et al., 2013).

Other markers of exposure or effects were also involved in the formation of the proteinaceous matrix that is integral to mollusk shell structure development. Temptin, a component of the tyrosinase metabolic pathway which is involved in larval shell formation (Liu et al., 2015) and insoluble shell matrix protein 5 appeared in the markers of exposure (Supplementary Table 1) in pooled larvae. They were not identified as markers of effect, so they are likely not directly involved in the abnormal development of larvae. Perlucin and perlucin-like protein (Weiss et al., 2000) were identified as markers of effect (4 copies) and exposure (2 copies) in single larvae, and markers of exposure in pooled larvae (Supplementary Table 1, 2, 5). *Pif* (Suzuki et al., 2009), on the other hand, was unique to the copper effects genes in pooled larvae (Supplementary Table 4 and Figure 9), and appeared as both a marker of effect (2 copies) and exposure (1 copy) in single larvae. Temptin, perlucin, and *pif*, along with several other shell matrix protein genes, were identified as markers of low-concentration copper exposure in *M. californianus* larvae (Hall et al., 2020). Sussarellu et al. (2018) examined the response of three different biomineralization genes (collagen, nacrein, and calcineurin B) to copper in early *C. gigas* larvae, and did not find a significant response, but we have similarly not identified these specific genes as copper responsive. We can thus conclude that specific shell matrix and biomineralization genes shell matrix pathways are targeted by copper in mussels, although possibly not in other bivalve larvae, and copper-induced abnormality may be associated with additional modulation of shell matrix protein forming genes.

While the cell adhesion GO term was only enriched among the markers of exposure in pooled larvae, there were still many genes related to the extracellular matrix and cell adhesion in both markers of exposure and effect in both pooled and single larvae (Supplementary Tables 1, 2, 4, 5). Cell adhesion is known to play an essential role in metazoan development, especially in nervous system development (Hynes and Lander, 1992), and a lack of proper cell adhesion mechanisms can lead to abnormal developmental patterns or embryo death (Gurdon, 1992). Previous research on oyster larval development found delayed and abnormal development in response to elevated CO_2_-induced expression of cell adhesion and extracellular matrix genes (De Wit et al., 2018). The prominence of cell adhesion genes among the markers of exposure is somewhat unexpected, as the literature suggests that disruption of cell adhesion often leads to abnormal development. However, there were unique cell adhesion genes that were identified as markers of effect, especially in the single larval markers of effect (e.g., multiple protocadherins present in markers of effect, vs. only a single copy in the markers of exposure - Supplementary Tables 2, 5), and some of the cell-adhesion-related markers of exposure (e.g., *POSTN*, *JAM2*, and *PCDH9*) were also shared amplitude-dependent markers of exposure and effect in pooled larvae (Supplementary Table 8). For these genes, higher expression was associated with abnormal development (Supplementary Table 8 and Figure 10). Therefore, it does appear that certain aspects of cell adhesion are involved in abnormal development induced at low copper concentrations, and that some cell adhesion genes can serve as good markers of effect.

This study also provides insight into the molecular mechanisms associated with natural abnormal development, which is still not well understood in molluscan systems. Genes that were DE in abnormal animals that weren’t exposed to copper represented functional categories similar to those identified in past studies of abnormal or delayed bivalve development. De Wit et al. (2018) assessed DE between larvae of oyster *C. gigas* that exhibited abnormal/delayed development in response to simulated OA and control larvae, and found that DE genes fell into four main categories: extracellular matrix, shell formation, transmembrane proteins, and protease inhibitors. At least several markers in each of these categories were identified in our gene sets as well, including some shared specific markers: caveolin, a gene with a thrombospondin motif, and a lectin (Supplementary Tables 9, 10). The differentially expressed cytoskeletal components in our study reflect previous findings that cytoskeletal component proteins, including tubulins, myosin, and tropomyosin, are differentially expressed between trochophore and D-hinge larvae of the oyster *C. gigas* (Huan et al., 2012). Huan et al. (2012) also identified cell proliferators as a key category of DE proteins, with several markers representing translation or ribosomes. We found several genes coding for DNA polymerases and DNA repair proteins (Supplementary Tables 9, 10), which could similarly be indicators of cell proliferation, but could also be indicative of DNA damage and DNA repair. Finally, previous research on *Pinctada fucata* (Pearl Oyster) transcriptional changes during development supports our finding that developmental genes are differentially expressed between D-hinge larvae and earlier stages prior to shell formation (Li et al., 2016).

Analysis of the phenotypic-anchored expression patterns revealed that while functional groups of sensitive transcriptional markers remain relatively consistent across sequencing approaches, trends in up or down regulation are less predictable. In the pooled sorted larval samples, the most sensitive markers were overwhelmingly upregulated in abnormal animals (Figures 7C–F). The single larvae markers of effect contained approximately equal numbers of genes that were upregulated and downregulated in abnormal larvae (Supplementary Table 5). In contrast, our previous study showed that genes that were downregulated were the most sensitive indicator of copper, with large-scale gene downregulation being a feature of the response to exposure to low copper concentrations (Hall et al., 2020). Furthermore, some of the sensitive upregulated markers in these experiments were only expressed at higher concentrations in our previous study. This shift in pattern can likely be attributed to differences in the nature of bulk pooled sequencing, sequencing of specific morphological groups, and sequencing of individual larvae. In both pooled and single larval samples, there were clear transcriptional differences associated with distinct morphologies. However, if those samples had been sequenced together, the nuances of morphology-specific expression would have been impossible to detect. At the lower copper concentration, 3 μg/l, there was consistently morphology-linked differential expression across both single larval and pooled larval dataset. However, the transcriptional profiles of normal and abnormal animals were not as reliably different at the higher copper concentration, 6 μg/l. Thus, it seems that pooled sequencing may be effective to detect biomarkers at higher concentrations, but that morphology-specific gene expression is more sensitive and informative at lower copper concentrations.

## Conclusion

We have identified robust transcriptional markers of copper exposure and effect in *M. californianus* larvae that could be used to improve the sensitivity and objectivity of bivalve embryo water quality assays for copper. Markers of effect were the most informative at lower copper concentrations, as substantial DE was consistently present in both sorted pools and single larval samples. We have also identified some biomarkers of copper exposure and effects that have not been previously identified in mussels. Markers of exposure exhibited similar functional categories to markers of effect, although often with intensified modulation or more gene copies activated in the markers of effect, which suggests that abnormal animals exercise similar yet amplified responses to copper, rather than modulating different responses and pathways. Markers of copper exposure and effect are characterized by genes involved in oxidoreductase activity, oxidative stress, cell adhesion, and extracellular proteinaceous matrix. The exact mechanisms of copper-induced abnormal development remain unclear, but these results highlight pathways that should be further explored at the enzymatic and cellular level.

## Data Availability Statement

The datasets presented in this study can be found in online repositories. The names of the repository/repositories and accession number(s) can be found below: https://www.ncbi.nlm.nih.gov/, PRJNA688298.

## Author Contributions

MH contributed to the experimental design, executed the experiments, analyzed the data, and authored the manuscript. AG contributed to the research idea and experimental design, executed the experiments, analyzed the data, and authored the manuscript. Both authors contributed to the article and approved the submitted version.

## Conflict of Interest

The authors declare that the research was conducted in the absence of any commercial or financial relationships that could be construed as a potential conflict of interest.

## Publisher’s Note

All claims expressed in this article are solely those of the authors and do not necessarily represent those of their affiliated organizations, or those of the publisher, the editors and the reviewers. Any product that may be evaluated in this article, or claim that may be made by its manufacturer, is not guaranteed or endorsed by the publisher.

## Footnotes

1
http://geneontology.org/page/download-ontology
