# Supplementary figures and images for: Single-Larva RNA Sequencing Identifies Markers of Copper Toxicity and Exposure in Early Mytilus californianus Larvae
Source: Front Physiol. 2021 Dec 9;12:647482. doi: 10.3389/fphys.2021.647482 (PMC8696127; doi:10.3389/fphys.2021.647482)

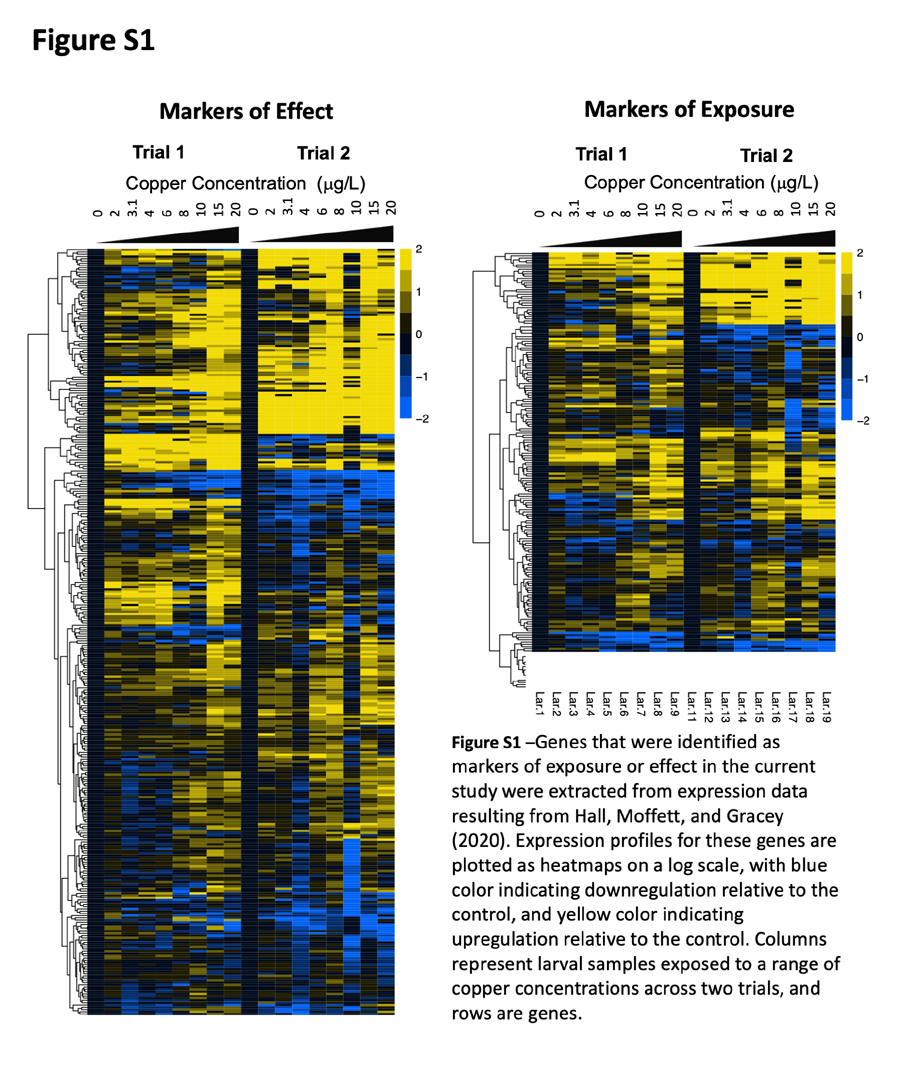

Supplement: Supplementary file 11 [file Image_1.TIFF]
